# Factors determining level of hospital care and its association with outcome after resuscitation from pre-hospital pulseless electrical activity

**DOI:** 10.1186/s13049-018-0568-0

**Published:** 2018-11-19

**Authors:** Sini SAARINEN, Ari SALO, James BOYD, Päivi LAUKKANEN-NEVALA, Catharina SILFVAST, Ilkka VIRKKUNEN, Tom SILFVAST

**Affiliations:** 1 FinnHEMS Research and Development Unit and Emergency Medical Service, FinnHEMS 30, Tampere University Hospital, University of Tampere, PO Box 2000, FI-33521 Tampere, Finland; 2 Emergency Medical Services, Department of Emergency Medicine, University of Helsinki and Helsinki University Hospital, Helsinki, Finland; 3 FinnHEMS Research and Development Unit, FinnHEMS Ltd, Vantaa, Finland; 4 University of Helsinki, Helsinki, Finland; 5 Helsinki University Hospital and University of Helsinki, Helsinki, Finland

**Keywords:** Heart arrest, Cardiopulmonary resuscitation, Pulseless electrical activity, Post-resuscitation care

## Abstract

**Background:**

Patients resuscitated from out-of-hospital cardiac arrest (OHCA) with pulseless electrical activity (PEA) as initial cardiac rhythm are not always treated in intensive care units (ICUs): some are admitted to high dependency units with various level of care, others to ordinary wards. Aim of this study was to describe the factors determining level of hospital care after OHCA with PEA, post-resuscitation care and survival.

**Methods:**

Adult OHCA patients with PEA (*n* = 221), who were resuscitated in southern Finland between 2010 and 2013 were included, provided patient survived to hospital admission. The patients were divided into four groups according to the level of hospital care provided: ordinary ward and Level 1–3 ICUs. Differences in patient characteristics, post-resuscitation care and survival were compared between the groups.

**Results:**

Most patients (62.4%) were treated at Level 2 ICUs. Longer time to ROSC and advanced age decreased admission rate to Level 2 or 3 post-resuscitation care, whereas good pre-arrest CPC (1–2) increased the admission rate to Level 2/3 ICUs independently. Treatment with targeted temperature management (TTM) (4.1%) or early coronary angiography (3.2%) were very rare. Prognostic decisions were made earlier in the lower treatment intensity groups (*p* < 0.01). One-year survival rate was 24.0, 17.1% survived with good neurological outcome. Neurological outcome was better with more intensive care. After adjustment, level of care was not independent predictor for outcome: only return of spontaneous circulation (ROSC) time, cardiac arrest cause and pre-arrest performance affected independently to 1-year survival, age and ROSC for neurologic outcome.

**Conclusions:**

PEA are usually admitted to Level 2 ICUs for post-resuscitation care in the capital area of Finland. Age, ROSC and pre-arrest CPC were independent predictors for level of post-resuscitation care. TTM and early CAG were rare and provided only for Level 3 ICU patients. Prognostication was earlier in lower level of care units. Good neurologic survival was more common with more intensive level of post-resuscitation care. After adjustment, level of care was not independent predictor for survival or neurologic outcome: only ROSC, cardiac arrest cause and pre-arrest performance predicted 1-year survival; age and ROSC neurologic outcome.

## Background

The proportion of pulseless electrical activity (PEA) as initial cardiac rhythm has increased during last decades, currently accounting for 19 to 35% of out-of-hospital cardiac arrest (OHCA) [1] [2] [3] [4]. Although patients resuscitated from PEA have a worse prognosis than those resuscitated from ventricular fibrillation (VF) or pulseless ventricular tachycardia (VT) [5], improvements in PEA patients’ survival rate have been reported [2] [6] [7] with 5.7–15.7% survival to hospital discharge [1] [2] [8] [9] [10] [11]. Improvement has been noted both in pre-hospital survival [6] [12] as well as in-hospital survival [6]. Investigators have speculated that use of targeted temperature management (TTM) is one of the reasons for increased in-hospital survival [5].

The European Resuscitation Council (ERC) recommends post-resuscitation care in the intensive care units (ICUs) and suggests that all resuscitated patients, irrespective of the initial cardiac rhythm, should be treated with TTM [13], and early coronary angiography (CAG) should also be considered [14]. However, due to limited ICU resources and a less favourable prognosis, all OHCA patients with PEA are not admitted to ICU: our earlier study revealed that 16% of Nordic ICUs do not usually admit patients with initial PEA or asystole (ASY) [15]. Patients not admitted to ICUs are treated in high dependency units with various levels of care or in ordinary wards. Further, the definition of an ICU varies around the world. Therefore, the task force of the World Federation of Societies of Intensive and Critical Care Medicine (WFSICCM) recently proposed a categorization of ICUs to Level 1 to Level 3 ICUs based on the care they provide (Table 1) [16].

**Table 1:** Classification of ICUs as purposed by WFSICCM. Modified from Marshall et al. 2017 [16]

	Level 1	Level 2	Level 3
Capacity	Short-term support of mild organ dysfunction	Basic support of organ dysfunction	Complex management of organ dysfunction
Treatment	Non-invasive respiratory support	Mechanical ventilator support, pharmacologic hemodynamic support, intermittent RRT	Advanced ventilator and hemodynamic support, continuous RRT
Monitoring	Non-invasive	Invasive	Advanced invasive
Personnel	Nurse patient ratio 1:4 or 1:3 Physicians with some experience on critical care available during day	Nurse patient ratio 1:3 or more Physicians with some ICU training during day, available at night	Nurse patient ratio 1:1 or 1:2 Physicians with ICU training on call 24/7

*ICU* intensive care unit, *RRT* renal replacement therapy, *WFSICCM* World Federation of Societies of Intensive and Critical Care Medicine

To our knowledge, factors associated with selection to different levels of care among PEA patients has not been described earlier. The aim of our study was to describe which clinical features were different between PEA patients selected for Level 1, 2 or 3 ICU treatment or treatment in the ordinary wards. We presumed that patients admitted to Level 3 ICUs were considered more likely to survive. In addition, two senior physicians, who were blinded to the survival outcome, estimated whether any of PEA patients treated in the ordinary wards could have benefited from intensive care.

## Methods

The patients (*n* = 221) included in this retrospective observational study were resuscitated by the Helsinki Emergency Medical Services (EMS’s) in Helsinki, Finland, or by the helicopter EMS (HEMS) of the Helsinki university hospital, FinnHEMS 10, in Southern Finland between 1 March 2010 and 31 December 2013. Both EMS’s are physician staffed, with most physicians specialized in anaesthetics and intensive care. All adult OHCA patients with PEA as initial cardiac rhythm were included, provided return of spontaneous circulation (ROSC) was achieved on the scene and the patient survived to hospital admission (*n* = 224).

Patients were identified from Helsinki EMS’s cardiac arrest registry and FinnHEMS10 pre-hospital database. Data on hospital treatment were retrieved from hospital patient records. Cerebral Performance Category (CPC) -classification [17] was used to evaluate pre-arrest cerebral performance. CPC-classification is a five-stage scale of neurological state, in which classes 1–2 correspond to sufficient cerebral function for independent activities for daily life, while classes 3–4 reflect dependency on others, class 5 means death. Neurological outcome is considered favorable with CPC score 1 or 2. Patients performance was also evaluated with Eastern Cooperative Oncology Group (ECOG) Performance Status (0 = fully active, 1 = physically strenuous activity restricted, able to do light work, 2 = independent on self-care, unable to work, 3 = limited self-care, in bed more than 50% of waking hours, 4 = completely disabled, confined to bed, 5 = dead) [18].

In addition, pre-arrest performance was categorized as in the FINNRESUSCI study [19] into four classes (1 = able to work or in equivalent condition, 2 = unable to work but independent in activities of daily life, 3 = requiring some help in daily activities, 4 = dependent on others for performing daily activities).

Patients were divided into four groups according to the level of care provided as categorized by the WFSICCM Task Force: patients treated at Level 1–2 ICUs, Level 3 ICUs and those treated in the ordinary wards (Table 1) [16]. In Finland, usually only Level 3 ICUs are called ICUs and Level 1–2 ICUs are often called high dependency units.

### Statistical analyses

The pre-arrest characteristics, resuscitation details, post-resuscitation care and survival of these four patient cohorts (Ward, Level 1 ICU, Level 2 ICU, Level 3 ICU) were compared. As age was symmetrically distributed, mean ages of the groups with standard deviation was reported, and 1-way Anova -test was used to compare group means of age. Multiple comparisons between group mean ages were analysed with Tukey HSD test. Normal distribution of response, ROSC and ventilator times were unrealistic based on Shapiro-Wilk’s test, thus medians with interquartile ranges (IQRs) were reported and independent samples Kruskal-Wallis test was used for group comparison. Pearson’s chi-square test was used for gender and cause of CA, which had nominal measurement scale. To all other variables of baseline characteristics, post-resuscitation and survival data with ordinary scale, Mantel-Haenszel test of trend was used.

To evaluate independent factors determining the Level of care, baseline factors and cardiopulmonary resuscitation (CPR) details were included in a logistic regression model for adjustment. For this model, ward and Level 1 ICU groups were combined, as well as Level 2 and 3 ICUs. Patients fully awake on hospital admission were excluded from the logistic regression model. For comparison of post-resuscitation care and survival, ward and Level 1 ICU groups were combined. Another logistic regression model was created to adjust factors determining good neurologic survival. For 1-year survival proportional hazards regression model was fitted. The post-resuscitation care and survival models included baseline characteristics, CPR details and Level of care as independent factors. Reducing of factors in these models was made by backward stepwise selection, based on the probability of the likelihood-ratio statistic.

Based on the data available on hospital admission (pre-arrest diagnoses, symptoms before OHCA, cerebral performance, independency on others and resuscitation details: witnessed CA, bystander CPR, EMS response time and time to ROSC), two senior anaesthesia and intensive care physicians (T.S and I.V) independently estimated each patient treated in ordinary wards or in Level 1 ICUs whether in their opinion there was an indication for more intensive (Level 2 or 3 ICU) treatment or not. The physicians were blinded to information about the treatment unit chosen, methods used, survival and neurological outcome. Estimation was carried out without pre-defined criteria for ICU-admission, since to our knowledge, no general pre-defined criteria for PEA patients’ admission is being used in Finland at the moment. Only those patients who both of the physicians independently thought would have benefited from more intensive care (Level 2–3) were included in this analysis. The inter-rater agreement was estimated by Cohen’s kappa. *P*-values of < 0.05 were considered to indicate significance. SPSS Statistics (IBM, Version 25) was used for data analysis.

Permission to conduct the study was granted by the Helsinki University Hospital Research and Development Division. Because this study used previously collected data, the researchers were not required to submit the study protocol for ethical board review.

## Results

### Selection of post-resuscitation care unit

During the study period, 224 adult patients were resuscitated from OHCA with PEA and admitted to hospital with sustained ROSC. Three patients taken directly to operation room for surgical intervention (ruptured aortic aneurysm) were excluded, thus 221 patients formed the study population. The most common units for post-resuscitation care were Level 2 ICUs (*n* = 138, 62.4%), followed by Level 3 ICUs (*n* = 39, 17.6%), ordinary wards (*n* = 28, 12.7%) and Level 1 ICU (*n* = 16, 7.2%).

The mean age of all patients was 64.0 ± 15.1 years (Table 2). Mean age differed significantly between the groups: compared to Level 3 ICU patients, ward patients were on average 13.4 years older (95%CI 4.1–22.6, *p* < 0.01), Level 1 ICU patients were 18.9 years older (95%CI 7.8–30.0, p < 0.01) and Level 2 patients were 6.8 years older (CI95% 0.02–13.6, *p* = 0.049). In addition, Level 1 ICU patients were on average 12.0 years older (CI95% 2.2–22.0) than Level 2 ICU patients (*p* = 0.01). Gender distribution of the patients did not differ between the groups.

**Table 2:** Baseline and clinical characteristics according to intensity of post-resuscitation care, n (%)

	All *n* = 221	Ward *n* = 28	Level 1 ICU *n* = 16	Level 2 ICU *n* = 138	Level 3 ICU *n* = 39	p
Age (y), mean ± SD	64.0 ± 15.1	70.1 ± 15.2	75.6 ± 7.8	63.5 ± 14.9	56.7 ± 14.2	< 0.001
Male	143 (64.7)	20 (71.4)	9 (56.3)	92 (66.7)	22 (56.4)	0.48
Pre-arrest diagnoses						
Hypertension	102 (46.4)	13 (46.4)	9 (56.3)	60 (43.8)	20 (51.3)	0.96
Coronary artery disease	44 (20.0)	8 (28.6)	5 (31.3)	29 (21.2)	2 (5.1)	0.02
Diabetes	57 (25.9)	5 (17.9)	9 (56.3)	37 (27.0)	6 (15.4)	0.43
Heart failure	28 (12.7)	7 (25.0)	3 (18.8)	16 (11.7)	2 (5.1)	0.01
Renal failure	15 (6.8)	3 (10.7)	1 (6.3)	9 (6.6)	2 (5.1)	0.39
Memory impairment	20 (9.1)	3 (10.7)	4 (25.0)	11 (8.0)	2 (5.1)	0.17
Other brain disease¤	37 (16.8)	6 (21.4)	4 (25.0)	23 (16.8)	4 (10.3)	0.18
Pre-arrest performance						0.02
1	138 (62.7)	15 (53.6)	6 (37.5)	89 (65.0)	28 (71.8)	
2	26 (11.8)	3 (10.7)	0 (0.0)	19 (13.9)	4 (10.3)	
3	54 (24.5)	10 (35.7)	10 (62.5)	28 (20.4)	6 (15.4)	
4	2 (0.9)	0 (0.0)	0 (0.0)	1 (0.7)	1 (2.6)	
Pre-arrest performance ECOG						0.001
0	88 (40.2)	7 (25)	3 (18.8)	53 (39.0)	25 (64.1)	
1	52 (23.7)	8 (28.6)	3 (18.8)	37 (27.2)	4 (10.3)	
2	26 (11.9)	3 (10.7)	0 (0.0)	18 (13.2)	5 (12.8)	
3	53 (24.2)	10 (35.7)	10 (62.5)	28 (20.6)	5 (12.8)	
Pre-arrest CPC						< 0.001
1	114 (52.1)	10 (35.7)	5 (31.3)	69 (50.7)	30 (76.9)	
2	57 (26.0)	8 (28.6)	1 (6.3)	41 (30.1)	7 (17.9)	
3	48 (21.9)	10 (35.7)	10 (62.5)	26 (19.1)	2 (5.1)	
Accomodation type						0.02
Home, independent	152 (71.0)	18 (66.7)	6 (37.5)	99 (74.4)	29 (76.3)	
Home, assisted	36 (16.8)	5 (18.5)	4 (25.0)	18 (13.5)	9 (23.7)	
Nursing home	26 (12.1)	4 (14.8)	6 (37.5)	16 (12.0)	0 (0.0)	
Resuscitation details						
Witnessed CA	193 (88.1)	26 (92.9)	14 (87.5)	115 (84.6)	38 (97.4)	0.79
Bystander CPR	70 (34.0)	9 (33.3)	2 (15.4)	44 (34.4)	15 (39.5)	0.41
Response time, median IQR	6 (0–9)	7 (1–9)	7 (0–9)	5 (0–9)	5 (0–8)	0.67
ROSC time, median (IQR)	18 (12–23)	17 (12–23)	22 (16–25)	18 (12–22)	17 (13–25)	0.63
Cause of CA						0.048
Cardiac	66 (30.3)	8 (28.6)	6 (37.5)	40 (29.6)	12 (30.8)	
Hypoxia	61 (28.0)	10 (35.7)	6 (37.5)	36 (26.7)	9 (23.1)	
Intoxication	16 (7.3)	0 (0.0)	0 (0.0)	11 (8.1)	5 (12.8)	
Neurological	21 (9.6)	4 (14.3)	4 (25.0)	12 (8.9)	1 (2.6)	
Other	24 (11.0)	2 (7.1)	0 (0.0)	20 (14.8)	2 (5.1)	
Unknown	30 (13.8)	4 (14.3)	0 (0.0)	16 (11.9)	10 (25.6)	

*ICU* intensive care unit, *CPC* cerebral performance category, *CA* cardiac arrest, *CPR* cardiopulmonary resuscitation, *ROSC* return of spontaneous circulation, *SD* standard deviation, *IQR* interquartile range, *ECOG* Eastern Cooperative Oncology Group

Response and ROSC times are presented in minutes, as median (IQR)

¤diagnoses of previous intracranial haemorrhage, stroke, contusion, severe congenital disability, brain atrophy, encephalitis, and brain tumour

See Methods for details of performance, ECOG and CPC classifications. *P*-values < 0.05 indicate significant difference between at least two Levels of care (age, response-and ROSC times) or significant trend in results when Level of care increases (other variables). Thus *P*-value < 0.05 in headings of pre-arrest performance, ECOG, CPC, accommodation type and cause of cardiac arrest indicates that there is significant difference between groups in at least one of the values (for example in amount of CPC 1, CPC 2 or CPC 3) and there is a trend according to increasing Level of care. If the resulting p-value is > 0.05, it means there is no significant difference between any of the values. Probability test used are explained in detail in the Methods section

The prevalence of pre-arrest diagnoses of coronary artery disease and cardiac failure differed between the groups (*p* = 0.02 and p = 0.01 for trend, respectively): coronary artery disease had the highest prevalence in the Level 1 ICU group, cardiac failure in the Ward group. Both diagnoses were the rarest among Level 3 ICU patients (Table 2). The cause of OHCA also differed between the groups, Level 3 ICU patients had less hypoxic and neurological causes (*p* = 0.048). No difference was found between the groups in the number of pre-arrest diagnoses of hypertension, diabetes, memory impairment/disorder or other brain disease and renal failure. Data on resuscitation details (witnessed OHCA, bystander CPR, EMS response time and time to ROSC) did not differ between the groups. Factors describing patients’ performance and dependence on others were all significantly different between the groups (Table 2). Patients in the Level 3 ICU group were most commonly in classes which stand for independency and good performance (Table 2).

In the logistic regression model, patients fully awake on hospital admission (*n* = 10) were excluded. Ward and Level 1 ICU were compared to Level 2 and 3 ICUs. After adjustment for other baseline factors (Table 2), longer time to ROSC and advanced age decreased the admission rate to Level 2 or 3 post-resuscitation care, whereas good pre-arrest CPC (1–2) increased Level 2/3 ICU admission rate (Table 3).

**Table 3:** Independent predictors for Level 2–3 admission (compared to ward/Level 1) (*n* = 211)

	p	OR	95% CI for OR
Age (y)	0.03	0.97	0.94–0.996
ROSC (min)	0.03	0.95	0.91–0.996
CPC			
1	0.04	2.69	1.07–6.78
2	0.01	4.50	1.41–14.35
3–4		1	

*OR* odds ratio, *CI* confidence interval, *ROSC* return of spontaneous circulation, *CPC* cerebral performance category

In the model, patients in CPC 1 or 2 were compared to patients in CPC 3–4 (reference). OR > 1 indicates that the probability of Level 2–3 ICU admission is higher than that for reference patients. *P* values were calculated using Wald’s test

According to the blinded assessment, in the opinion of both physicians, 16 patients (36.4%) admitted to ward or Level 1 ICU could have benefited from Level 2–3 intensive care. The two physicians’ opinions differed from each other in 10 (22.7%) patients, indicating moderate agreement (Cohen’s kappa = 0.548).

### Post-resuscitation care

Nine patients (4.1%) were treated with TTM, all in Level 3 ICUs (Table 4). Early CAG was provided for 7 patients (3.2%) and later during hospital stay for 6 patients (2.7%). Coronary artery bypass graft surgery and renal replacement therapy were very rarely provided. The median time on ventilator was longest 35 h (18-67 h) in Level 3 ICU group. Level 3 patients had the longest ventilator treatment times, but no difference existed between Level 2 and Level 3 ventilator treatment times (*p* = 0.06). The decision whether to continue active post-resuscitation care of a comatose patient was made earlier in the lower treatment intensity groups (*p* < 0.01). Prognostic decisions were made < 24 h after OHCA in 87 (61.7%) patients and up to 93.5% in ward or Level 1 ICU treated patients (Table 4).

**Table 4:** Post-resuscitation care in treatment groups, n (%)

	All n = 221	Ward /Level 1 ICU (*n* = 44)	Level 2 ICU (n = 138)	Level 3 ICU (n = 39)	p
TTM	9 (4.1)	0	0	9 (23.1)	< 0.01
CAG < 48 h	13 (5.9) 7 (3.2)	1 (2.3) 0	9 (6.5) 4 (2.9)	3 (7.7) 3 (7.7)	0.69
CABG	6 (2.7)	1 (2.3)	4 (2.9)	1 (2.6)	0.93
Time on ventilator (h)	18 (4–41)	3 (0–12)	20 (6–46)	35 (18–67)	< 0.01
Brain CT	110 (50.5)	14 (31.8)	72 (53.3)	24 (61.5)	0.01
NSE	64 (29.4)	2 (4.5)	49 (36.3)	13 (33.3)	< 0.01
Prognostication					< 0.01
< 24 h	87 (61.7)	29 (93.5)	51 (54.8)	7 (41.2)	
24-72 h	36 (25.5)	2 (6.5)	26 (28.0)	8 (47.1)	
> 72 h	18 (12.8)	0	16 (17.2)	2 (11.8)	

*ICU* intensive care unit, *TTM* targeted temperature management, *CAG* coronary angiography, *CABG* Coronary artery bypass graft surgery, *CT* computer tomography, *NSE* neuron specific enolase. Time on ventilator is presented as median (IQR). Probability tests used are explained in Methods section. P-value < 0.01 for prognostication indicates that there is significant difference between the groups in at least one of the prognostication time categories and there is a trend according to increasing level of care

### Survival and neurological outcome

Of all study patients, 72 (32.6%) survived to hospital discharge, 62 (28.1%) survived 90 days and 53 (24.0%) survived 1 year after OHCA. Patients treated in Level 3 ICU had highest 90-days and 1-year survival rates (Table 5), but no significant difference was found in 90-days and 1-year survival between the groups. Good neurological survival (CPC 1–2) 1 year after OHCA was reported in 37 (17.1%) patients. PEA patients treated in units providing more intensive post-resuscitation care had better neurological outcome: 11 (28.2%) Level 3 ICU patients, 22 (16.1%) Level 2 ICU and 4 (9.8%) ward/Level 1 ICU patients had CPC 1–2 1 year after OHCA (*p* = 0.02).

**Table 5:** Survival and neurological outcome according to the level of post-resuscitation care, n (%)

Survival	All n = 221	Ward /Level 1 ICU (n = 44)	Level 2 ICU (n = 138)	Level 3 ICU (n = 39)	p
90 days	62 (28.1)	11 (25.0)	39 (28.3)	12 (30.8)	0.56
1 year	53 (24.0)	8 (18.2)	33 (23.9)	12 (30.8)	0.18
CPC at 1 year					0.02
1	23 (10.6)	0	15 (10.9)	8 (20.5)	
2	14 (6.5)	4 (9.8)	7 (5.1)	3 (7.7)	
3	12 (5.5)	1 (2.4)	10 (7.3)	1 (2.6)	
4	0	0	0	0	
5	168 (77.4)	36 (87.8)	105 (76.6)	27 (69.2)	

*ICU* Intensive care unit, *CPC* cerebral performance category. P-value < 0.02 for CPC at 1 year indicates that there is significant difference between the groups in at least some of the categories (CPC 1–5) and there is a trend according to increasing level of care

After adjustment for baseline characteristics and CPR details, Level of care (ward/Level1 or Level 2 or Level 3) did not independently affect on 1-year survival or neurological outcome. Independent predictors for 1-year mortality were long time to ROSC, neurological reason for OHCA compared to cardiac reason and poor pre-arrest performance. For poor neurological outcome or death, advanced age and long time to ROSC were independent predictors (Table 6).

**Table 6:** Independent predictors of 1-year mortality and poor CPC at 1-year (n = 221)

1-year mortality	p	HR	95% CI for HR
ROSC (min)	< 0.01	1.04	1.02–1.06
Neurologic versus cardiac OHCA	< 0.01	1.92	1.19–3.08
Performance class 3–4 versus class 1	0.03	1.50	1.04–2.20
CPC 3–5 at 1 year	p	OR	95%CI for OR
Age (y)	< 0.01	1.05	1.02–1.08
ROSC (min)	< 0.01	1.17	1.10–1.26

*CPC* cerebral performance category, *HR* hazard rate, *OR* odds ratio, *CI* confidence interval, *ROSC* return of spontaneous circulation, *OHCA* out-of-hospital cardiac arrest

## Discussion

In this study on the level of post-resuscitation care and its’ association with outcome, we found that age, ROSC and pre-arrest CPC were independent predictors for selection of post-resuscitation care level. Good neurologic survival was more common with more intensive post-resuscitation care. After adjustment, level of care was not independent predictor for survival or neurologic outcome: only ROSC, cardiac arrest cause and pre-arrest performance affected independently to 1-year survival, age and ROSC for neurologic outcome.

To our knowledge, factors influencing PEA patients’ admission to post-resuscitation care units have not been described earlier. Among our PEA patients, Level 2 ICUs were the common units for post-resuscitation care and Level 3 ICU care was provided for only 17.6% of patients. Patients selected for more intensive care were younger, had less coronary artery disease and cardiac failure, less neurologic and hypoxic cause for CA and more favourable pre-arrest performance and CPC. A favourable CPC category, short time to ROSC and young age were independent factors associated with admission to more intensive level of care.

We assumed that patients admitted to care at wards or Level 1 ICUs were considered to have either desperate prognosis, which cannot be improved with more intensive care, or so good prognosis, that outcome is good even without Level 2 or 3 care. As this was assumed to confuse the analysis of factors affecting admission to different levels of care, patients awake at hospital admission were excluded. Our results support the hypothesis that patients considered to survive are more likely admitted to more intensive care, since all independent predictors have been reported to associate with increased survival [20] [21]. Nursing home residents have been reported to have worse survival rates [20], which is in line with our findings on the effect of CPC on Level 2–3 ICU admission. A previous study of all-rhythm witnessed OHCA showed worse outcomes in patients with pre-arrest diagnoses of hypertension, diabetes, myocardial infarction and congestive heart failure [22]. Advanced age is associated with worse outcomes in CA in patients more than 65 years of age [20], but there is a wide variation in the effect of age on outcomes, with some studies showing no association between the two [23]. Of CPR details, witnessed CA [5] [12] [24], bystander CPR [5] [12], short first response time [12] and short time to ROSC [21] are known to be associated with improved prognosis at the population level.

According to the opinion of the blinded physicians in this study, more than a third of the patients treated outside Level 2–3 ICUs would possibly have benefited from intensive care. Moderate agreement between the two physicians indicates that patient selection is an ambiguous process: information about the patient’s pre-arrest condition is often limited, and prognostication at the early phase of post-resuscitation care is challenging, since reliable prognosis of neurological recovery is possible only 3 days after CA [25]. The decision whether to admit patients resuscitated from PEA to an ICU often needs to be made immediately after hospital admission. Further, the decision whether to transfer a PEA patient to a Level 3 ICU or to a local hospital with Level 1–2 ICU should optimally be made already on the scene. Without predefined criteria, the experience and opinions of the treating physician influence on the decision of Level 2 or 3 ICU admission and may lead to alternating patient selection. On the other hand, predefined criteria may prevent individual variation but may also exclude potential survivors who do not fit the criteria. Scoring systems to predict OHCA patients’ outcome and therefore advocate Level 2–3 ICU treatment are not commonly used, although some prediction tools have been created [24] [26] [27]. Thus, implementing a reliable and tested scoring system could help clinicians in decision making.

TTM (4.1%) and early CAG (3.2%) were provided very rarely and only for Level 3 ICU patients. The initial rhythm in OHCA has been reported to greatly affect post-resuscitation care: up to 39% of UK ICUs have reported they never use TTM for patients resuscitated from PEA or ASY [28]. 54–87% of ICUs have reported to usually cool patients resuscitated from VF/VT, but only 28–30% usually cool patients resuscitated from PEA or ASY [28]. In addition, early CAG is provided for 37–58% of VF/VT patients compared to 7–16% of patients resuscitated from PEA or ASY [29] [30]. However, CAG is a specific treatment for acute coronary syndrome, which is more common in VF/VT patients. This study does not define which of the PEA patients had possible acute coronary syndrome and thereby potential to benefit from CAG. The heterogeneity of provided post-resuscitation care reflects in part the inconclusive evidence regarding optimal post-resuscitation care. RCT-based evidence on efficacy of TTM in non-shockable OHCA patients is still lacking [31]. In addition, efficacy of early CAG among OHCA patients comes from observational studies [32]. The use of TTM could increase the survival rates: in observational studies, up to 2.9 times increased odds for survival with good neurological outcome have been reported for non-shockable patients [33] [34]. Categorical exclusion of PEA patients from TTM, because of initial cardiac rhythm, can result in exclusion of some patients who may benefit from this treatment.

Compared to lately reported PEA patients’ hospital discharge rates of 5.9–15.7% [1] [2] [8] [9] [10] [11], results of our study are encouraging: 32.6% of patients admitted to hospital survived to hospital discharge and 24% survived 1 year, 17.1% with good neurology. Survival rates increased by increasing level of care post-resuscitation care, but the difference was insignificant between the groups. Survival with good neurology increased significantly with more intensive level of care. However, after adjustment, level of care was not independently associated with survival or neurological outcome. There is a possibility that the study population was too small to show an association between with the level of care and outcome. In addition, including patients awake on admission, who probably did not need that intensive care, in this analysis could conflict the results. On the other hand, Guidet et al. reported in a recently published multicentre, cluster-randomized clinical trial, that among critically ill elderly patients (over 75 years), promotion of ICU admission did not reduce 6-month mortality nor improve functional status or physical quality of life 6 months after [35]. In our population, the independent predictors of good outcome (young age, short time to ROSC, cardiac arrest cause and good pre-arrest performance) have been reported to improve outcome also in earlier studies [20] [21] [36] [37].

Two thirds of the patients who do not survive to discharge after OHCA die as a result of hypoxic-ischemic brain injury, and approximately half of the patients die after withdrawal of life-sustaining treatment after a bad outcome is predicted [38] [39]; thus, the timing of prognostication is crucial. The ERC recommends postponing prognostic decisions to > 72 h after OHCA in comatose patients [40]. In the present study, the prognostic decision was made early (< 24 h) in most of the patients (62%) and significantly earlier in the lower level of care groups. However, a more detailed review showed that 62 (28.1%) of patients needed help in activities of daily life or lived in nursing homes and 5 had a do not attempt resuscitation (DNAR) order, of which prehospital paramedics and physicians were not aware of. Resuscitation was attempted, and ROSC was achieved in patients with a severe medical history and high dependency on others, in whom a DNAR decision probably should have been made before OHCA. Still, it is possible, that among some patients, the prognostic decision and the decision to withdraw life-sustaining treatment were made too early, based on unreliable indicators of poor outcome.

This study was limited by its retrospective nature. Furthermore, patients were resuscitated and treated in Southern Finland, in the capital area and around it. These results cannot be generalized to concern the rest of the country. Although the hospitals follow the same international guidelines, ICU and high dependency unit selection criteria might vary between different regions and hospitals due to regional policies and varying ICU capacity. As data were collected retrospectively, researchers estimated patients’ performance and CPC status according to patients records, which could lead to false evaluation. In addition, there were relatively small number of patients in ward, Level 1 and 3 ICU groups.

## Conclusions

Based on the present findings, we conclude that only a minority (17.6%) of OHCA patients with PEA are admitted to Level 3 ICUs for post-resuscitation care in the capital area of Finland. Age, ROSC and pre-arrest CPC were independent predictors for selection of post-resuscitation care level. TTM (4.1%) and early CAG (3.2%) were rare and provided only for Level 3 ICU patients. Prognostication was earlier in lower level of care units. Good neurologic survival was more common with more intensive level of post-resuscitation care. After adjustment, level of care was not independent predictor for survival or neurologic outcome: only ROSC, cardiac arrest cause and pre-arrest performance affected independently to 1-year survival, age and ROSC for neurologic outcome.

## Abbreviations

ASY: Asystole
CA: Cardiac arrest
CABG: Coronary artery bypass graft surgery
CAG: Coronary angiography
CI: Confidence interval
CPC: Cerebral performance category
CPR: Cardiopulmonary resuscitation
CT: Computer tomography
DNAR: Do not attempt resuscitation
ECOG: Eastern Cooperative Oncology Group
EMS: Emergency medical service
ERC: European Resuscitation Council
HA: Hazard rate
HEMS: Helicopter emergency medical service
ICU: Intensive care unit
IQR: Interquartile range
NSE: Neuron specific enolase
OHCA: Out-of-hospital cardiac arrest
OR: Odds ratio
PEA: Pulseless electrical activity
RCT: Randomized controlled trial
ROSC: Return of spontaneous circulation
RRT: Renal replacement therapy
SD: Standard deviation
TTM: Targeted temperature management
VF: Ventricular fibrillation
VT: Ventricular tachycardia
WFSICCM: World Federation of Societies of Intensive and Critical Care Medicine
